# Identification and validation of potential common biomarkers for papillary thyroid carcinoma and Hashimoto’s thyroiditis through bioinformatics analysis and machine learning

**DOI:** 10.1038/s41598-024-66162-2

**Published:** 2024-07-06

**Authors:** Hui Jiang, Yanbin He, Xiaofeng Lan, Xiang Xie

**Affiliations:** 1 Department of Ultrasound, The Second Affiliated Hospital of Anhui Medical Universty, Hefei, 230601 Anhui China; 2 Department of Interventional Ultrasound, The Second Affiliated Hospital of Anhui Medical Universty, Hefei, 230601 Anhui China; 3 Dian Diagnostics Group Co., Ltd, Hangzhou, 310000 Zhejiang China; 4 Key Laboratory of Digital Technology in Medical Diagnostics of Zhejiang Province, Hangzhou, 310030 Zhejiang China

**Keywords:** Cancer, Computational biology and bioinformatics, Immunology, Biomarkers, Endocrinology, Pathogenesis

## Abstract

There is a growing body of evidence suggesting that Hashimoto’s thyroiditis (HT) may contribute to an increased risk of papillary thyroid carcinoma (PTC). However, the exact relationship between HT and PTC is still not fully understood. The objective of this study was to identify potential common biomarkers that may be associated with both PTC and HT. Three microarray datasets from the GEO database and RNA-seq dataset from TCGA database were collected to identify shared differentially expressed genes (DEGs) between HT and PTC. A total of 101 genes was identified as common DEGs, primarily enriched inflammation- and immune-related pathways through GO and KEGG analysis. We performed protein–protein interaction analysis and identified six significant modules comprising a total of 29 genes. Subsequently, tree hub genes (CD53, FCER1G, TYROBP) were selected using random forest (RF) algorithms for the development of three diagnostic models. The artificial neural network (ANN) model demonstrates superior performance. Notably, CD53 exerted the greatest influence on the ANN model output. We analyzed the protein expressions of the three genes using the Human Protein Atlas database. Moreover, we observed various dysregulated immune cells that were significantly associated with the hub genes through immune infiltration analysis. Immunofluorescence staining confirmed the differential expression of CD53, FCER1G, and TYROBP, as well as the results of immune infiltration analysis. Lastly, we hypothesise that benzylpenicilloyl polylysine and aspirinmay be effective in the treatment of HT and PTC and may prevent HT carcinogenesis. This study indicates that CD53, FCER1G, and TYROBP play a role in the development of HT and PTC, and may contribute to the progression of HT to PTC. These hub genes could potentially serve as diagnostic markers and therapeutic targets for PTC and HT.

## Introduction

Hashimoto’s thyroiditis (HT) is a prevalent autoimmune condition characterized by lymphocyte infiltration into the thyroid gland and the generation of autoantibodies targeting antigens specific to the thyroid^1,2^. It is well-known that chronic inflammation and autoimmune diseases increase the risk of malignancy^3,4^. Among thyroid cancers, papillary thyroid carcinoma (PTC) is the most common and its incidence is increasing^5,6^. The prevalence of concurrent HT in PTC patients is approximately 33%, and there is an increasing trend in the co-occurrence of PTC and HT^7,8^. HT and PTC are both thyroid disorders that share certain clinical and histopathological features. The relationship between HT and PTC was first described by Dailey et al. in 1955, highlighting the association between HT and the occurrence and development of PTC^9^. Additional research has continued to reinforce this connection, indicating that HT might act as a stand-alone contributing element to the development of PTC^10,11^.

In the development of HT-induced PTC, the process of inflammation establishes an advantageous setting for the formation of malignancy. This conversion is influenced by various cytokines and growth factors, which impact the reactivity of the stromal cells and ultimately result in the malignant transformation of the epithelial cells^12^. Furthermore, tumor-infiltrating immune cells may contribute to abnormal DNA repair, ultimately inducing PTC^13^. The specific molecular mechanisms underlying the transformation of HT into PTC remain unclear. Consequently, researchers are increasingly focused on identifying the drivers and potential mechanisms involved in the progression from HT to thyroid malignancy.

Therefore, identifying the key genes that are shared by HT and PTC can shed light on the common molecular pathways that contribute to the development and progression of these conditions. Such knowledge could potentially inform the development of targeted therapies and interventions that specifically address these shared genetic factors.

Through bioinformatics, a wide range of data can be analyzed, including genetic sequences, protein structures, and gene expression patterns. In recent years, the field of bioinformatics has seen extensive use in analyzing diagnostic and therapeutic targets for a range of diseases^14,15^. Machine learning (ML) algorithms provides a valuable approach for mining and predicting patterns from complex datasets^16^. By training algorithms on existing data, they can learn patterns and make accurate predictions on new data. Furthermore, The utilization of ML algorithms has gained popularity in the identification of potential biomarkers, aiding in the prompt identification of various disorders.

**Figure 1:**
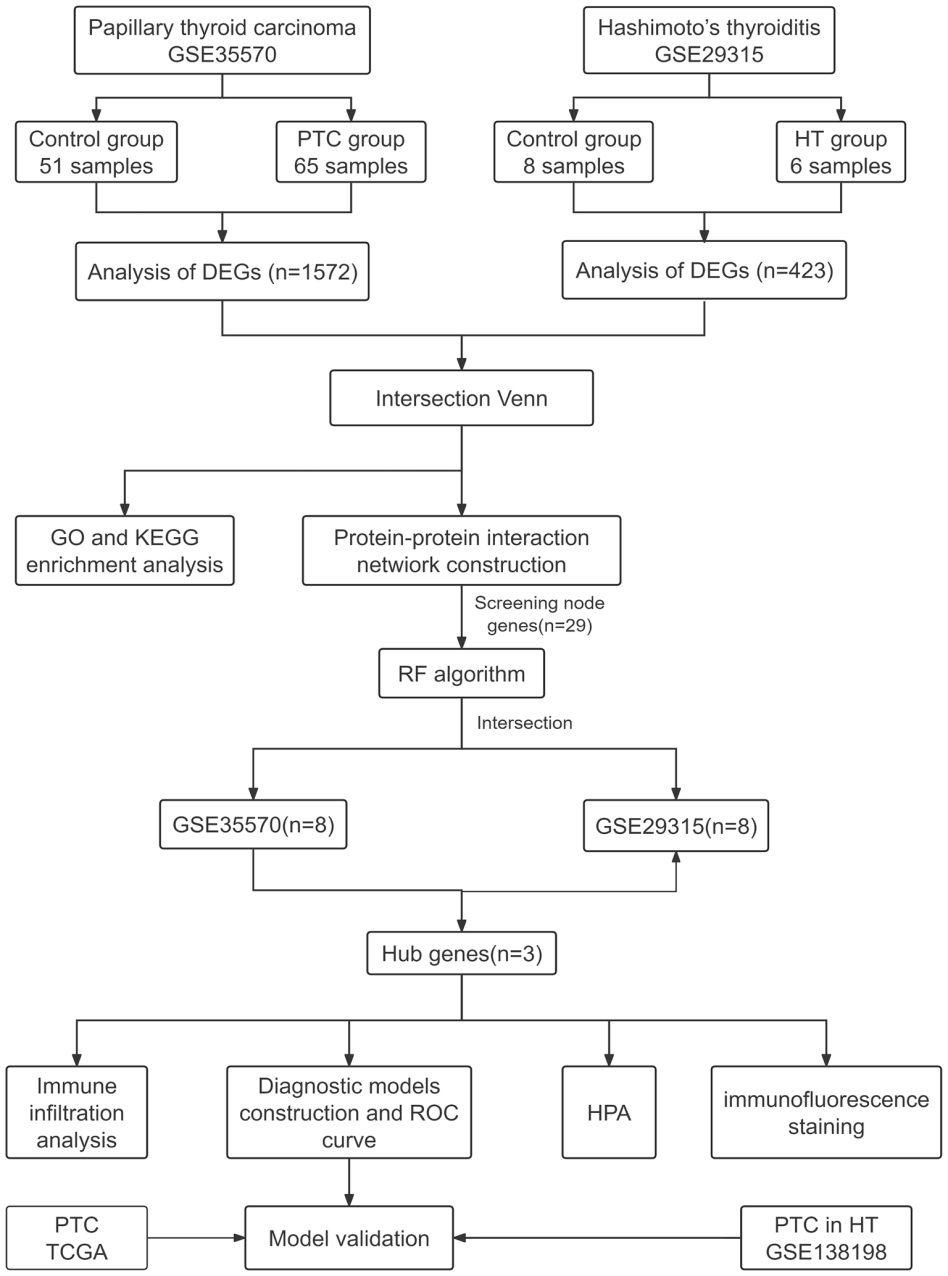
Flow chart of the whole study.

In this study, bioinformatics analysis and machine learning techniques were employed to analyze gene expression profiles and identify biomarker candidates. We also utilized artificial neural network (ANN), EXtreme Gradient Boosting(XGBoost) and Decision Tree(DT) to develop tree diagnostic models. Moreover, we observed various dysregulated immune cells that were significantly associated with the hub genes through immune infiltration analysis. Immunofluorescence (IF) staining confirmed the differential expression of CD53, FCER1G, and TYROBP, as well as the results of immune infiltration analysis. The results suggest that CD53, FCER1G, and TYROBP are involved in the biological process of HT and PTC, offering insights for early diagnosis and new therapeutic targets. Please refer to Fig. 1 for the flow chart.

## Materials and methods

### Microarray data

In our analysis, the microarray datasets GSE35570 and GSE29315 were obtained from the Gene Expression Omnibus (GEO) database (https://www.ncbi.nlm.nih.gov/geo/)^17^. GSE35570 (GPL570) consists of gene expression profiles from 65 PTC samples and 51 normal tissues. The GSE29315 (GPL8300) dataset includes 6 HT samples and 8 control samples. Additionally, the TCGA dataset and GSE138198 (GPL6244) dataset were also downloaded as external validation sets for the key gene expression. The TCGA dataset contains RNA-seq data from 53 samples of PTC and 37 samples of normal thyroid tissue. The GSE138198 contains 8 PTC in HT samples and 3 normal samples. The samples from GSE35570 were randomly divided into a training set (80%) and a test set (20%) to train and validate the diagnostic models for PTC. We utilized the TCGA dataset to evaluate the model’s diagnostic efficacy for PTC. Additionally, we utilized the GSE138198 dataset to evaluate the model’s diagnostic efficacy for PTC in HT.

### Identification of differential genes

Expression matrix and clinical information were obtained from the GEO database using the ‘GEOquery’ R package. The probesets were annotated using the ‘AnnoProbe’ package, and probes without corresponding gene symbols were filtered out. Additionally, genes with multiple probe groups were averaged. We conducted an analysis on the differentially expressed genes (DEGs) by utilizing the ‘get_deg_all’ function within the ‘tinyarray’ package in R. Following this, the ‘tinyarray’ package was utilized to create heatmaps, PCA plots, and volcano plots. DEGs were recognized by applying a criterion of a log2-fold change (|log2FC|) of at least 1 and an adjusted p-value below 0.05. The Venn diagram was used to determine the common DEGs with the same trend in PTC and HT.

### Functional enrichment analysis

Gene Ontology (GO)^18^ employs biological processes (BP), cellular components (CC), and molecular functions (MF) to annotate gene properties and gene items. The Kyoto Encyclopedia of Genes and Genomes (KEGG)^19^ serves as a notable open database that supplies annotation data regarding gene signal transduction and disease pathways. The ‘clusterProfiler’ R package was utilized to carry out the enrichment analysis of differentially expressed genes. A statistically significant threshold was established at an adjusted p-value of less than 0.05.

### Protein–protein interaction (PPI) analysis and module analysis

The STRING database (https://string-db.org/)^20^ was used to analyze protein interactions, including both physical and functional interactions. A protein–protein interaction (PPI) network was created with a minimum required interaction score of 0.4. The results were visualized using Cytoscape software, and we calculated and analyzed the degree centrality (DC) topological properties for each node in the PPI network using the CytoNCA plug-in in Cytoscape software^21^. To identify hub targets, we used DC ≥ 2 × median DC as the screening criteria^22^. After that, the Minimal Common Oncology Data Elements (MCODE) plug-in was utilized to identify the genes involved in the interactions. The ClueGO plug-in in Cytoscape software was used to To further analyze the GO enrichment of genes in the most important module. By utilizing ClueGO, we can effectively explore the functional significance of genes within these modules and understand their involvement in biological processes. It enables a more detailed and comprehensive investigation, enhancing the overall understanding of the gene sets in question.

### Screening and evaluation of common hub genes in PTC and HT

The RF algorithm was employed to identify potential genes for diagnosing PTC and HT in two datasets, GSE35570 and GSE29315. The RF algorithm ranked the genes based on their importance, and we compared the gene importance rankings between the two datasets. The top eight ranked genes that were common to both datasets were considered candidate hub genes for diagnosing PTC and HT. Furthermore, we evaluated the diagnostic value of each hub gene using receiver operating characteristics (ROC) analysis. To further validate the diagnostic potential of these hub genes, we assessed their performance in diagnosing PTC using the TCGA, and evaluated their performance in diagnosing PTC with HT using the GSE138198.

### Diagnostic model construction and feature importance analysis

Three ML diagnostic model (ANN, XGBoost and DT) were developed using the R package ‘tidymodels’, based on the genes identified by RF. Bayesian optimization was employed for hyperparameter tuning of the diagnostic models. The performance and models generation were evaluated through five-fold stratified cross-validation. ROC analysis was utilized to assess the diagnostic performance and accuracy of the prediction models. The Shapley Additive exPlanations (SHAP) method was utilized to interpret the optimal model by visualizing the contribution of each gene to the diagnosis of PTC. SHAP is a visualized approach for interpreting the output of a machine learning model. A positive SHAP value indicates a positive effect of the gene characteristic on the occurrence of disease, while a negative value indicates a negative effect.

### Analysis of protein expression

The current database of the Human Protein Atlas (HPA) (https://www.proteinatlas.org/)^23^ contains more than 26,000 antibodies that have undergone professional verification through immunohistochemical staining. IHC-stained images of the proteins associated with hub genes were collected from the HPA database for PTC and normal thyroid tissues. The aim was to investigate any differences in protein expression of hub genes between these two sample types.

### Analysis of immune infiltration

To assess the variances in immune cell infiltration between PTC, HT and normal samples, we employed the CIBERSORT algorithm^24^.The significance of these differences was evaluated using the Wilcoxon test, and the results were visualized through box plots. Moreover, the connection between immune cells and hub genes was examined through Spearman correlation analysis, and the outcomes were presented via heat maps.

### Immunofluorescence staining

During the period between January 2022 and December 2022, a total of 10 patients diagnosed with PTC accompanied by HT were enrolled in the research. The specimens, consisting of paraffin-embedded tissues, were obtained from the Department of Pathology at the Second Affiliated Hospital of Anhui Medical University. The expression levels of specific proteins, namely CD53 (Affinity, Jiangsu, China), FCER1G (Affinity, Jiangsu, China), and TYROBP (Affinity, Jiangsu, China), were examined using IF analysis. IF staining was also employed to detect the infiltration of immune cells. The expression levels of Cd4 (Abcam, Cambridge, UK), Cd8 (Abcam, Cambridge, UK), and Cd86 (Abcam, Cambridge, UK) were assessed to determine the infiltration status of CD4^+^ T cells, CD8^+^ T cells, and macrophages, respectively. Briefly, paraffin-embedded thyroid tissue was cut into 5 μm thick sections. The slides were then dewaxed and rehydrated. Epitope retrieval was performed by boiling the slides in citrate antigen retrieval solution (PH = 6) (Zsbio, Beijing, China) for 3 min. After cooling, the slides were rinsed three times with phosphate buffered saline (PBS) for 5 min each time. Proteins were then blocked with 5% bovine serum albumin (BSA) for 30 min. Tissue sections were incubated with the primary antibody overnight at 4 °C and then treated with the secondary antibody for 1 h at room temperature. Finally, the nuclei were stained with 4,6-diamidino-2-phenylindole (DAPI) (Beyotime, Shanghai, China) and the slides were blocked using an antifade mounting medium (Beyotime, Shanghai, China). The slides were promptly examined under an fluorescence microscope (Nikon, Tokyo, Japan). Image J software was employed to evaluate the . mean fluorescence intensity (MFI) of three randomly selected areas on each tissue slide. This investigation was conducted with the approval of the Ethics Committee at the Second Affiliated Hospital of Anhui Medical University. In accordance with the ethical principles outlined in the Declaration of Helsinki, all participants provided informed consent before participating in the study. All the methods were conducted in strict adherence to the relevant guidelines and regulations.

### Analysis of potential therapeutic agents

The Drug-Gene Interaction Database DGIdb (https://www.dgidb.org/)^25^ is a comprehensive database that consolidates information on drug-gene interactions from various sources such as organizations, presentations, databases, and web resources. In our study, we utilized the DGIdb database to analyze a list of carefully screened core genes. Our aim was to predict the drugs that target the core genes involved in HT and PTC pathogenesis, as well as obtain drug-gene interaction scores. Subsequently, we can identified the most promising therapeutic drugs based on these scores.

### Ethics approval and consent to participate

Human samples protocols obtained approval from the Institutional Research Ethics Committee at the Second Affiliated Hospital of Anhui Medical.

## Results

### Identify shared differential genes

**Figure 2:**
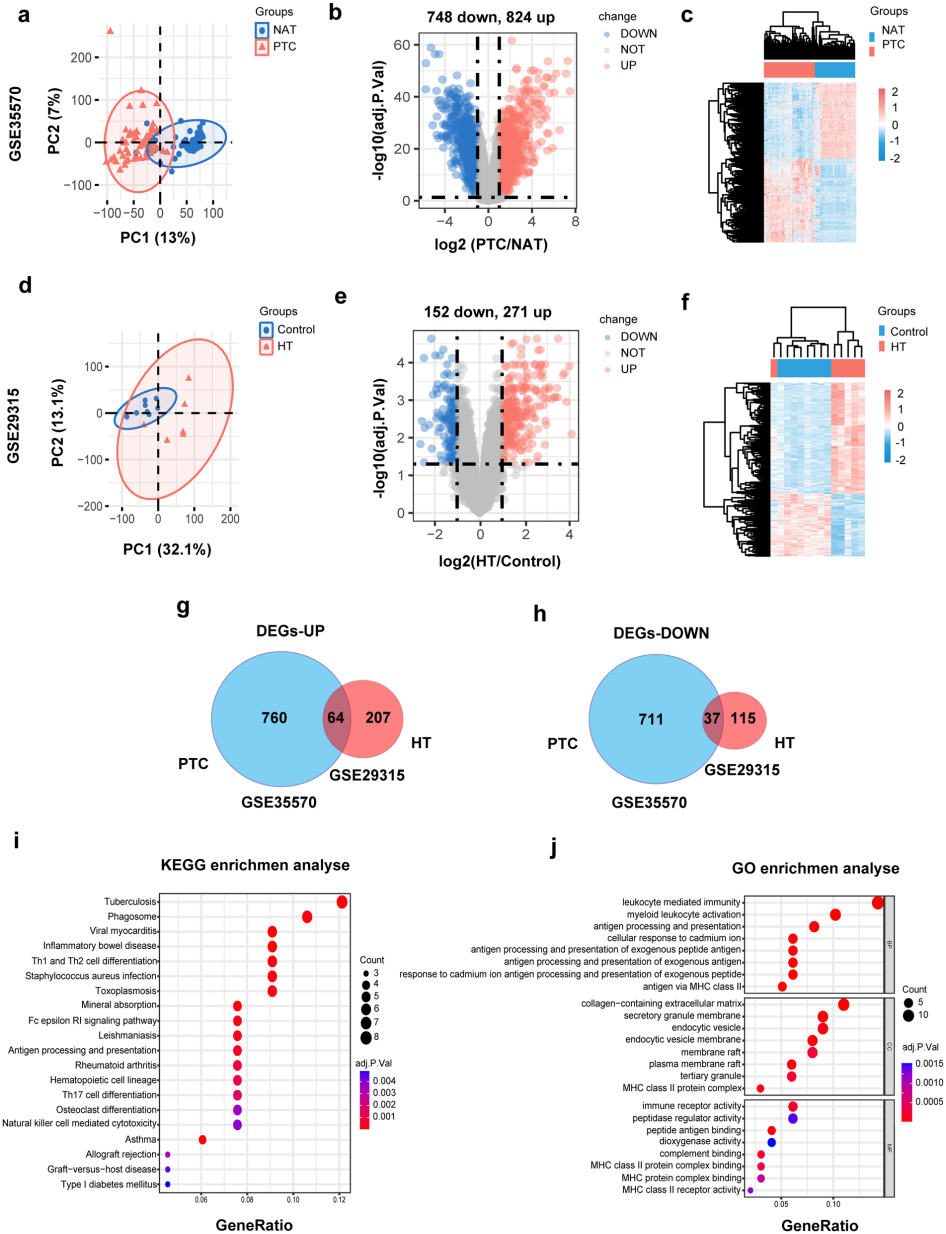
Differential expression gene analysis, function enrichment analysis and pathway enrichment analysis. (**a**) The PCA plot of GSE35570. (**b**, **c**) The Volcano plot and heatmap of DEGs in GSE33570. (**d**) The PCA plot of GSE29315. (**e**, **f**) The Volcano plot and heatmap of DEGs in GSE29315. (**g**) Venn plot of the up-regulated DEGs. (**h**) Venn plot of the down-regulated DEGs. (**i**) The KEGG enrichment analyses of DEGs. (**j**) The GO enrichment analyses of DEGs.

When conducting PCA analysis on the expression matrices of GSE33570 (Fig. 2a) and GSE29315 (Fig. 2d), we observed a clear two-sided distribution of samples in both the disease group and the control group. In the analysis of the GSE35570 dataset, a total of 1572 distinct genes were detected as being differentially expressed. These DEGs were categorized into 824 up-regulated genes and 748 down-regulated genes (Fig. 2b). Similarly, we observed 423 DEGs in the GSE29315 dataset, including 271 up-regulated DEGs and 152 down-regulated DEGs (Fig. 2e). Next, the GEGs of the two datasets are displayed heatmaps for both datasets (Fig. 2c,f). Furthermore, we employed a Venn diagram to identify the overlapping genes with the same directional trend, resulting in 64 genes being up-regulated (Fig. 2g) and 37 genes being down-regulated (Fig. 2h).

### Functional analysis of shared differential genes

In order to enhance our comprehension of the fundamental biological functions linked to the 101 DEGs, an assessment of GO and KEGG enrichment was conducted using the ‘clusterProfiler’ software package in R. An analysis of GO highlighted that these shared genes were mainly enriched in leukocyte mediated immunity, myeloid leukocyte activation, and antigen processing and presentation (Fig. 2j). Additionally, the DEGs exhibited significant enrichment across the top five KEGG pathways, including Tuberculosis, Phagosome, Viral myocarditis, Inflammatory bowel disease, and Th1 and Th2 cell differentiation (Fig. 2i). Apparently, the functions of differentially expressed genes are closely associated with the immune function of the body. The core genes primarily serve the purpose of activating immune cells.

### PPI network establishment and module examination

To carry out the PPI analysis, we utilized the STRING online tool and visualized the outcomes using the Cytoscape software (Supplementary Fig. S1a). The PPI network showed 68 nodes and 498 edges. The DC value of each node was calculated, with a median value of 11. Based on this, we identified 17 hub genes of PPI network: TYROBP, ITGB2, STAT1, HLA-DRA, C1QB, MMP9, FCER1G, IL10RA, LCP2, LY86, CD53, CD14, CD163, HCK, MNDA, HLA-DPA1, and ALOX5AP. Subsequently, we employed the MCODE plug-in to identify six modules (Supplementary Fig. S1b,c), which included a total of 29 common DEGs. These DEGs were LCP2, TYROBP, CD53, LY86, ITGB2, FCER1G, MNDA, C1QB, HCK, IL10RA, HLA-DRA, ALOX5AP, MT1G, MT1F, MT1E, MT1X, ISG15, IFIT3, PSMB9, GBP2, CD14, CD163, VSIG4, CAV1, TIMP1, S100A4, SDC2, FGFR2, and STAT1. The most important module comprises 12 genes (LCP2, TYROBP, CD53, LY86, ITGB2, FCER1G, MNDA, C1QB, HCK, IL10RA, HLA-DRA, ALOX5AP), which were further analyzed using the ClueGO plug-in in Cytoscape software. The investigation revealed that these genes primarily function in activating neutrophils to participate in the immune response and activating innate immunity (Supplementary Fig. S1d).

### Diagnostic value of shared hub genes

**Figure 3:**
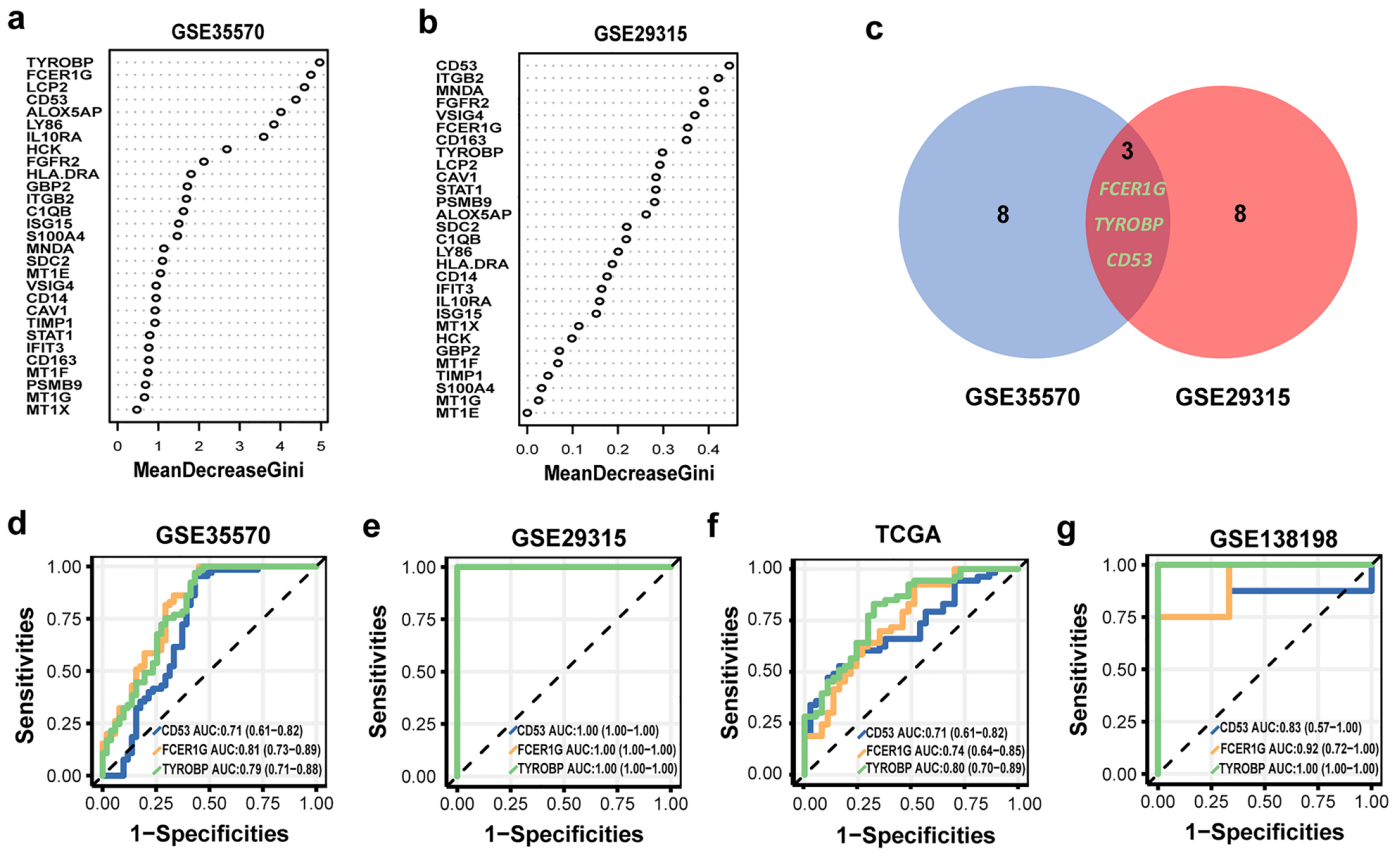
Screening of hub genes and the diagnostic value of hub genes. (**a**) The rankings of gene importance in GSE35570. (**b**) The rankings of gene importance in GSE29315. (**c**) Venn plot of the top eight genes in GSE35570 and GSE29315. (**d**) Diagnostic value of hub genes in the GSE35570. (**e**) Diagnostic value of hub genes in the GSE29315, (**f**) Diagnostic value of hub genes in the TCGA. (**g**) Diagnostic value of hub genes in the GSE138198.

In this study, we analyzed a total of 26 genes from six modules extracted from MCODE. To determine the importance of each gene, we employed the RF algorithm in two datasets, namely GSE35570 (Fig. 3a) and GSE29315 (Fig. 3b). By comparing the rankings of gene importance in both datasets, we identified the top eight genes that were consistently ranked highly. To visualize this overlap, we created a Venn diagram (Fig. 3c), which revealed three genes (CD53, FCER1G and TYROBP) that were shared between the two datasets. Remarkably, these three genes overlap with the hub genes identified through the PPI analysis based on DC values, as well as the genes found in the most significant module. These three genes showed promising diagnostic potential for HT and PTC. To evaluate the diagnostic value of the common hub genes, we computed the Cutoff Value, sensitivity, specificity, AUC and 95% CI for each gene in the four datasets (Table 1). In the GSE35570 dataset (Fig. 3d), the AUC values were as follows: CD53 (AUC 0.71, 95% CI 0.61–0.82), FCER1G (AUC 0.81, 95% CI 0.73–0.89), and TYROBP (AUC 0.79, 95% CI 0.71–0.88). In the GSE29315 dataset (Fig. 3e), the AUC values were as follows: CD53 (AUC 1.00, 95% CI 1.00–1.00), FCER1G (AUC 1.00, 95% CI 1.00–1.00) and TYROBP (AUC 1.00, 95% CI 1.00–1.00). In the TCGA dataset (Fig. 3f), we validated the diagnostic value of the common hub genes for PTC. The AUC values were as follows: CD53 (AUC 0.71 95% CI 0.61–0.82), FCER1G (AUC 0.74, 95% CI 0.64–0.89) and TYROBP (AUC 0.80, 95% CI 0.70–0.89). To further evaluate the diagnostic value of the common hub genes for PTC in HT, we computed the AUC and 95% CI for each gene using GSE1398198. In the GSE138198 dataset (Fig. 3g), the AUC values were as follows: CD53 (AUC 0.83, 95%CI 0.57–1.00), FCER1G (AUC 0.92, 95% CI 0.72–1.00) and TYROBP (AUC 1.00, 95% CI 1.00–1.00). We also analyzed the difference box plots between the two groups in the four datasets (Supplementary Fig. S2). Our analysis using box plots revealed a noteworthy disparity in gene expression between the HT group and the control group in GSE29315. This disparity serves as an explanation for the AUC values of the three hub genes in GSE29315, all of which were observed to be 1.

**Table 1:** AUC analysis of hub genes in the GSE35570, GSE29315, TCGA and GSE138198.

ID	Gene	Cutoff value	Sensitivity	Specificity	AUC	95% CI
	CD53	6.04	0.95	0.57	0.71	0.61–0.82
GSE35570	FCER1G	4.41	1.00	0.55	0.81	0.73–0.89
	TYROBP	6.43	0.97	0.57	0.79	0.71–0.88
	CD53	6.35	1.00	1.00	1.00	1.00–1.00
GSE29315	FCER1G	5.38	1.00	1.00	1.00	1.00–1.00
	TYROBP	7.62	1.00	1.00	1.00	1.00–1.00
	CD53	4.15	0.53	0.84	0.71	0.61–0.82
TCGA	FCER1G	4.22	0.93	0.49	0.74	0.64–0.85
	TYROBP	4.95	0.83	0.68	0.80	0.70–0.89
	CD53	8.71	0.75	1.00	0.83	0.57–1.00
GSE138198	FCER1G	7.65	0.75	1.00	0.92	0.72–1.00
	TYROBP	8.76	1.00	1.00	1.00	1.00–1.00

### Diagnostic model construction and feature importance analysis

**Figure 4:**
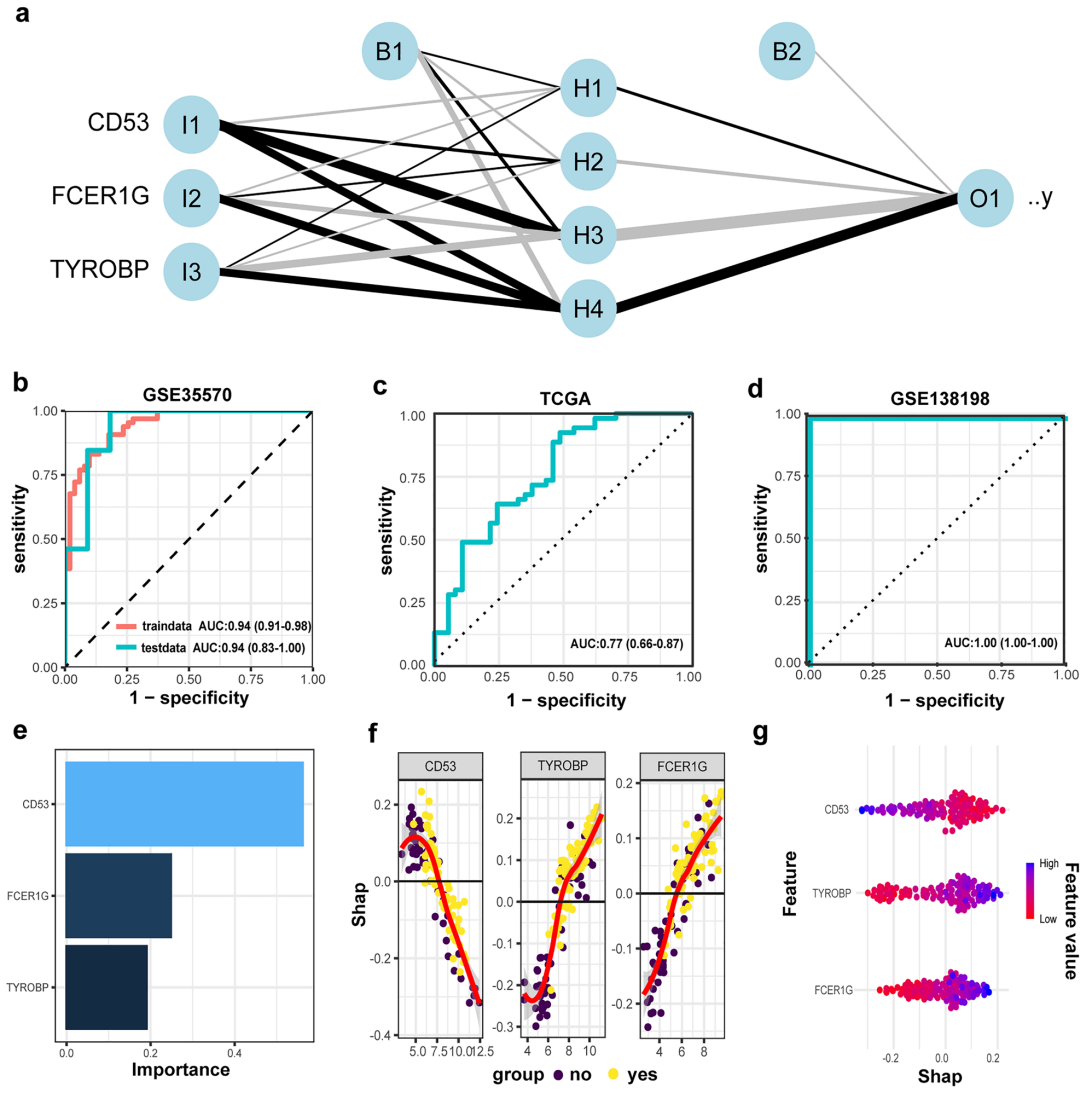
ANN model construction and feature importance analysis. (**a**) The ANN was constructed based on the shared hub genes. (**b**) Diagnostic value of the ANN model in the GSE35570. (**c**) Diagnostic value of the ANN model in the TCGA. (**d**) Diagnostic value of the ANN model in the GSE138198. (**e**) A score calculated by SHAP was used for each input feature. (**f**, **g**) Distribution of the impact of each feature on the full model output estimated using the SHAP values.

By using the GSE35570 dataset, we developed three diagnostic model specifically for PTC, incorporating these pivotal genes that were identified through our analysis. The ANN model (Fig. 4a) had 4 hidden units, a penalty of 0.0108, and was trained for 537 epochs. The ANN model achieved an AUC of 0.94 (95% CI 0.91–0.98) in the training set, while in the test set, the AUC was 0.94 (95% CI 0.83–1.00) (Fig. 4b). The XGBoost model had 8 mtry, 6 min_n, 3 max_depth, 0.001 learn_rate, and 0.07 loss_reduction and 0.97 sample_size. The XGBoost model achieved an AUC of 0.84 (95% CI 0.75–0.93) in the training set, while in the test set, the AUC was 0.62 (95% CI 0.42–0.83) (Supplementary Fig. S3a). The DT model had 0.0003 cost_complexity, 5 tree_depth and 6 min_n. The DT model achieved an AUC of 0.93 (95% CI 0.90–0.97) in the training set, while in the test set, the AUC was 0.83 (95% CI 0.65–1.00) (Supplementary Fig. S3b). Supplementary Table S1 displays the predictive performance of three machine learning models. The results indicate that the ANN model outperformed the other models, leading us to choose the ANN model for further analysis. TCGA dataset as external validation dataset was utilized to assess the diagnostic performance of the ANN model for PTC, yielding an AUC value of 0.77 (95% CI 0.66–0.87) (Fig. 4c). The GSE138198 dataset was used to evaluate the ANN model’s diagnostic efficacy for PTC in HT. In the GSE138198 dataset (Fig. 4d), the ANN model demonstrated a perfect AUC of 1.00 (95% CI 1.00–1.00). To provide clinicians with a better understanding of variable contributions, we utilized the SHAP algorithm to interpret the ANN prediction results. Figure 4e, f, g illustrated how the attributed importance of features changed as their values varied. Our findings reveal that CD53 had the most significant impact on the output of the ANN model. Initially, it was positively associated with the risk of PTC and then became negatively correlated after a turning point of approximately 6. TYROBP and FCER1G showed a positive correlation with the occurrence of PTC.

### Validation of common DEGs

**Figure 5:**
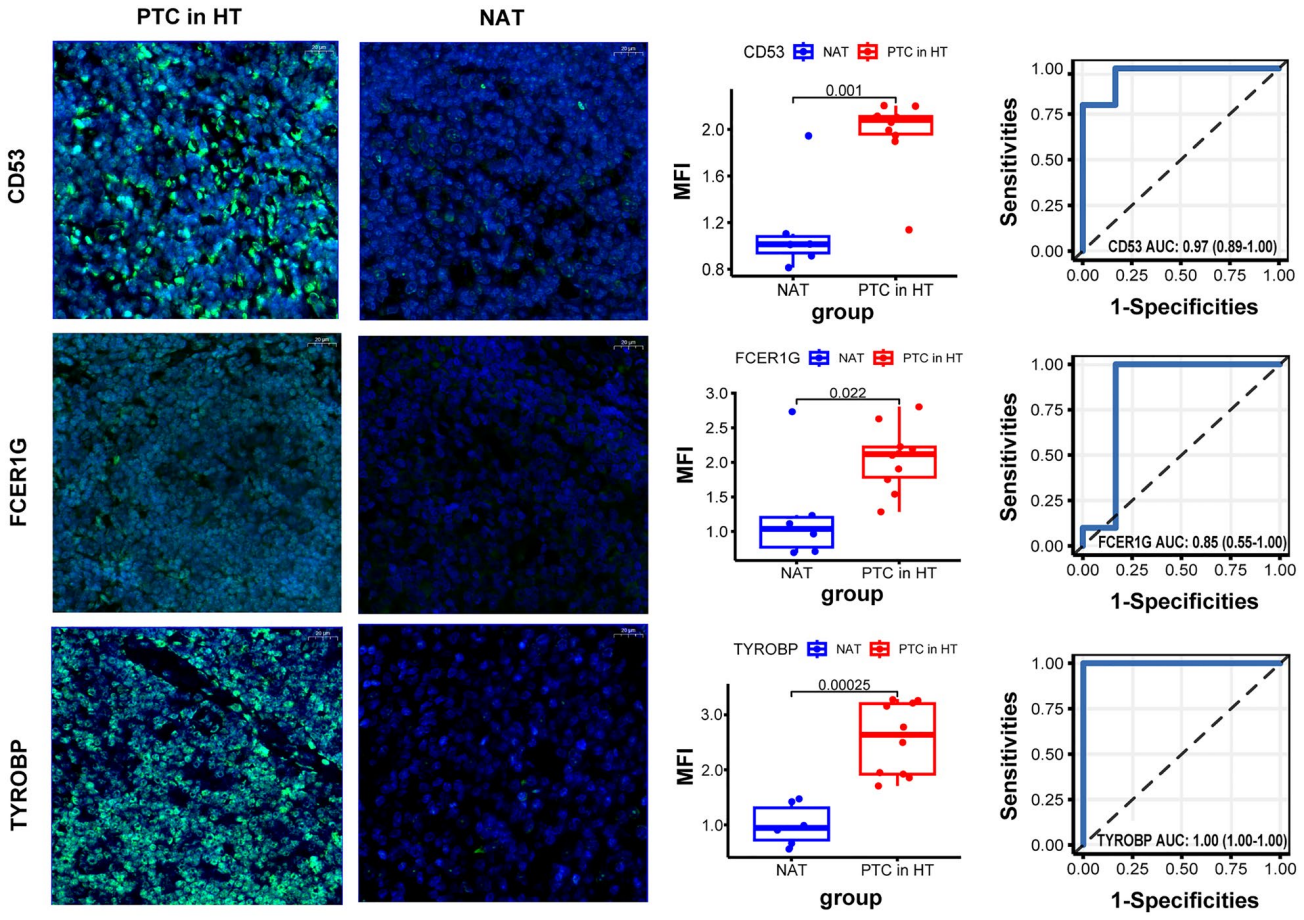
Microscopy scan of IF staining showed the distribution of CD53(green), FCER1G(green), and TYROBP(green), in HT-related PTC tissues and normal tissues adjacent to the tumour (NAT); as well as diagnostic value of CD53, FCER1G and TYROBP. MFI: Mean Fluorescence Intensity.

We analyzed the protein expression of the hub genes based on the HPA database (Supplementary Fig. S4). CD53 was highly expressed in both tumor and normal tissues, while FCER1G and TYROBP showed higher expression in tumors compared to normal tissues. Furthermore, IF staining was performed to measure the expressions of CD53, FCER1G, and TYROBP in our clinical samples, including 10 HT-related PTC tissues and 6 NAT. By performing IF analysis (Fig. 5), we obtained semi-quantitative results indicating significantly elevated fluorescence signal intensities for CD53, FCER1G, and TYROBP in the HT-related PTC group, as compared to the NAT group (*P* < 0.05).

### Analysis and validation of immune infiltration

**Figure 6:**
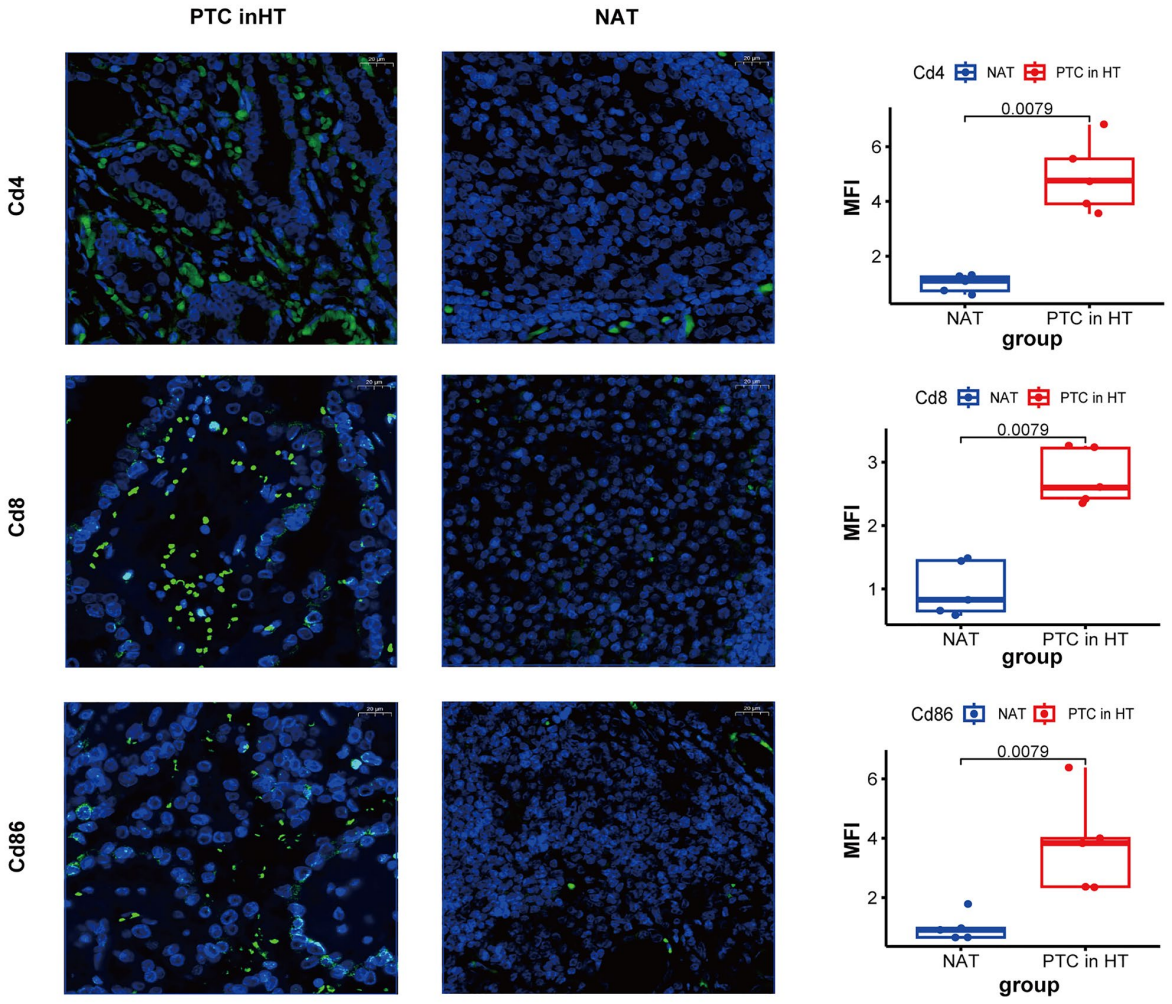
Microscopy scan of IF staining showed the distribution of Cd4(green), Cd8(green), and Cd86(green), in HT-related PTC tissues and normal tissues adjacent to the tumour (NAT). MFI: Mean Fluorescence Intensity.

Considering the important roles of immune and inflammatory responses in the development of HT and PTC, we analyzed the differences in immune cell infiltration patterns between PTC, HT and normal samples using the CIBERSORT algorithm. By utilizing the GSE35570 dataset, we identified 12 immune subgroups that exhibited significant variations between PTC and normal samples (Supplementary Fig. S5a). Additionally, the analysis of the GSE29315 dataset revealed 5 immune subgroups that were significantly different between HT and normal samples (Supplementary Fig. S5b). Among these, 4 common immune subpopulations were found to be significantly higher in both PTC and HT samples compared to normal samples. These subpopulations included T cells CD8, T cells CD4 memory resting, macrophages M1 and mast cells resting. Additionally, we conducted spearman correlation analysis between hub genes and immune cells (Supplementary Fig. S5c,d). The results suggested that immune responses could potentially contribute to the involvement of hub genes in PTC and HT progression. IF staining was utilized to identify immune cell infiltration in 5 cases of PTC in HT tissues and 5 cases of NAT (Fig. 6). The expression levels of CD4 + T-cell marker Cd4, CD8 + T-cell marker Cd8, and macrophage marker Cd86 were found to be significantly higher in the PTC in HT group compared to the NAT group. The IF staining results provided some extent of verification for the accuracy of the immune infiltration analysis results.

### Potential therapeutic agents for HT and PTC

Based on the three core genes screened in the RF algorithm, we conducted a search in the DGIdb database for relevant potential drugs. The results showed that only FCER1G had relevant drugs, while no relevant drugs were found for CD53 and TYROBP. FCER1G was predicted to have two potential drugs: benzylpenicilloyl polylysine and aspirin. Among these, benzylpenicilloyl polylysine had the highest score of 29.49, while aspirin had a score of only 1.26. We hypothesise that benzylpenicilloyl polylysine and aspirin may be effective in the treatment of HT and PTC and may prevent HT carcinogenesis.

## Discussion

Several studies have suggested a potential association between PTC and HT^9,10^. Patients with HT have been found to have a significantly higher incidence of PTC compared to those without HT^26^. However, some scholars argue that the co-existence of HT and PTC may be coincidental rather than causative^27^. The exact mechanism underlying this co-existence remains unclear. Therefore, it is crucial to identify common characteristics of these two diseases, as it may aid in the discovery of new diagnostic markers and treatment targets for patients with PTC and HT. Furthermore, such research may contribute to the prevention of cancer in individuals with HT.

Therefore, it is crucial to assess the associations between HT and PTC. In our study, we identified a total of 101 genes as common DEGs, which were primarily enriched in immunity and inflammation-related pathways according to GO and KEGG enrichment analysis. Furthermore, immune infiltration analysis revealed the involvement of various dysregulated immune cells in PTC and HT. The IF staining results provided some extent of verification for the accuracy of the immune infiltration analysis results. The classical view posits that the primary mediator of HT development and injury is the over-activation of CD8 + natural killer T cells by CD4 + T cells. As inflammation intensifies, CD8 + T cells are continuously recruited, resulting in an abnormal local CD4/CD8 ratio. This imbalance affects the immune microenvironment and may contribute to tumorigenesis^28^. However, there is a growing belief among scholars that the pathogenesis of HT begins with the infiltration of dendritic cells (DCs). These cells process specific antigens and present antigenic information to CD8 + T killer cells, which in turn activate and transform into cytotoxic T lymphocytes (CTLs). These CTLs exert their cytotoxic effects, leading to immunocide. This process is followed by an enhanced thyroid-specific immune response. The enhanced thyroid-specific immune response results in widespread infiltration of lymph node cells and a weakened immune surveillance function, potentially causing mutation of proto-oncogenes and the emergence of PTC^29^. Simultaneously, DCs induce the differentiation of CD4 + T helper cells into Th1 cells, which promote inflammation by releasing chemokines and cytokines. The number of Th1 cells increases and surpasses that of Th2 cells, resulting in an imbalance in the ratio of these two cell types. Ultimately, this imbalance leads to the development of the immune disease HT. Furthermore, DCs mediate the development of PTC by inducing abnormal differentiation of CD4 + T cells^30^. Additionally, regulatory T cells (Tregs) play a crucial immunosuppressive role in extracellular stabilization by inhibiting the activation and proliferation of the Th1 cell subpopulation of CD4 + T lymphocytes. Research has shown that a decrease in the number of Treg lymphocytes in patients with HT leads to an increase in the number of Th1 cells, resulting in an immune imbalance that contributes to the development of PTC^31^.It is evident that that immune and inflammatory responses play a critical role in the development of HT and PTC, with the recruitment and infiltration of immune cells promoting their progression^32,33^.

Based on 101 common genes, we employed PPI analysis and RF algorithms to identify three hub genes of PTC and HT, namely CD53, FCER1G, and TYROBP. These hub genes exhibit significant diagnostic value in the diagnosis of HT, PTC, and PTC in HT. In addition, we developed three diagnostic models (ANN, XGBoost and DT) for PTC using three hub genes. The ANN model has shown excellent performance in diagnosing PTC. We further utilized SHAP to interpret the features of the diagnostic model. Our model demonstrates excellent diagnostic performance not only in diagnosing PTC but also in identifying PTC in HT. Simultaneously, we validated the expression of three genes (CD53, FCER1G, and TYROBP) in clinical specimens using immunofluorescence staining, which were consistent with the HPA database. CD53, a prominent member of the tetraspanin family, exhibits high levels of expression in B cells, myeloid cells, and T cells^34,35^. While CD53 plays critical and distinctive roles within the immune system^36,37^, its involvement in tumorigenesis and its contribution to PTC development remain ambiguous, with only limited studies conducted thus far.TYROBP, on the other hand, is a tyrosine kinase binding protein that exhibits significant expression levels in various cancers. Extensive research has indicated a strong association between TYROBP and tumor progression^38^. Furthermore, TYROBP exhibits potential as both a diagnostic biomarker and a target for immunotherapy in cases of PTC^39^. However, further examination and investigation are required to fully comprehend its mechanism of action in PTC development.The High-affinity IgE receptor gamma subunit gene (FCER1G) represents a protein coding gene that is located on chromosome 1q23.3^40^. Previous studies have established FCER1G as a gene linked to innate immunity and its involvement in the malignant progression of conditions such as eczema, meningioma, PTC and childhood leukemia^41–43^. In this study, we discovered a strong association between these central genes and immune cell infiltration in HT and PTC. This suggests that candidate biomarkers may play a role in the development of HT and PTC by interacting with inflammatory immune pathways. However, their specific roles in cancer development remain unclear. We look forward to conducting further in-depth investigations to better understand their roles.

ML models, which are built on complex algorithms capable of analyzing and processing large amounts of data, have proven to be invaluable in improving the accuracy and efficiency of diagnosis^44,45^. These models enable healthcare professionals to make more informed decisions and provide patients with appropriate treatment plans. By utilizing machine learning models in disease diagnosis, we have the potential to reduce the need for invasive procedures and unnecessary tests. In our study, we developed three diagnostic models for PTC, of which the ANN model has shown excellent performance in diagnosing PTC. Furthermore, when we applied the model to diagnose PTC in HT, we observed that the model outperformed independent biomarkers, demonstrating its superior diagnostic value.

This study screened two potential drugs, benzylpenicilloyl polylysine (BPO) and aspirin, in treating HT and PTC. Aspirin is a widely used non-steroidal anti-inflammatory drug (NSAID) that offers analgesic, antipyretic, and anti-inflammatory effects^46^. It functions by inhibiting the enzyme cyclooxygenase, which reduces the production and release of inflammatory mediators. Aspirin is commonly prescribed for various diseases including rheumatoid arthritis, ankylosing spondylitis, and gout, among others^47^. Additionally, research has indicated that aspirin can provide pain relief for patients with Hashimoto’s thyroiditis^48^. BPO, a synthetic complex of benzylpenicillin polymerised with lysine, is commonly employed in skin allergy testing, particularly for patients with penicillin allergy^49^. The patient’s skin reaction is observed after injecting BPO under the skin. If the patient experiences redness, swelling, itching, or other allergic reactions following the BPO injection, it confirms an allergic reaction to penicillin^50,51^. It is crucial to note that BPO is currently solely used for allergy testing and not for treating the disease. Hence, further research is warranted to explore the potential application of BPO in thyroid diseases.

There are some limitations in our research. Primarily, the data utilized in this research primarily stemmed from gene expression data obtained through microarray technology, potentially overlooking certain genes that could be significant in PTC and HT. Microarray results are susceptible to various factors like experimental conditions and hybridization efficiency, which can lead to inaccurate quantitative outcomes. Furthermore, integrating bioinformatics with clinical practice is crucial for advancing precision medicine research and implementation. The vast amount of data produced by bioinformatics encompasses information across multiple levels such as the genome, transcriptome, proteome, among others. Effectively analyzing this data to extract valuable medical insights is a pressing issue in the realm of bioinformatics. The application of bioinformatics technology spans various stages, making it challenging to directly compare and validate results across different studies. Enhancing the reliability and reproducibility of bioinformatics technology is a critical challenge in clinical applications. Finally, due to the limited number of samples of PTC combined with HT in the TCGA and GEO databases, further validation is required to confirm the value of the three hub genes in diagnosing PTC in HT. In subsequent studies, we plan to build upon this research by conducting verification on a larger sample size.

## Conclusion

Our study utilized bioinformatics analysis and machine learning algorithms to identify four immune-related candidate hub genes (CD53, FCER1G, and TYROBP). Additionally, we developed three diagnostic models for PTC, of which the ANN model has shown excellent performance. We also observed a correlation between hub genes and immune cell infiltration. Furthermore, we analyzed the protein expression of these hub genes based on the HPA database. To validate these findings, we performed IF staining on clinical specimens. These results lay the foundation for early diagnostic strategies and potential therapeutic targets for patients with HT and PTC.

### Supplementary Information

Supplementary Information.

## Data Availability

The datasets provided in this investigation can be located in GEO database and TCGA database.
