# Supplementary material for: Identification and validation of potential common biomarkers for papillary thyroid carcinoma and Hashimoto’s thyroiditis through bioinformatics analysis and machine learning
Source: Sci Rep. 2024 Jul 6;14:15578. doi: 10.1038/s41598-024-66162-2 (PMC11227570; doi:10.1038/s41598-024-66162-2)
Supplement: Supplementary file 1 — Supplementary Information. [file 41598_2024_66162_MOESM1_ESM.docx]

Identification and Validation of potential common biomarkers for papillary thyroid carcinoma and Hashimoto's thyroiditis through Bioinformatics Analysis and Machine Learning

Hui JIANG^1^, Yanbin He^3,4^, Xiaofeng LAN^1^, Xiang XIE^1,2*^

^1^Department of Ultrasound,The Second Affiliated Hospital of Anhui Medical Universty,Hefei, 230601,Anhui,China

^2^Department of Interventional Ultrasound, The Second Affiliated Hospital of Anhui Medical Universty,Hefei, 230601,Anhui,China

^3^Dian Diagnostics Group Co., Ltd., 310000 Hangzhou, Zhejiang, China

^4^Key Laboratory of Digital Technology in Medical Diagnostics of Zhejiang Province, 310030 Hangzhou, Zhejiang, China

***To whom correspondence should be addressed.
Emial:** [sonographer@126.com](mailto:sonographer@126.com)

**
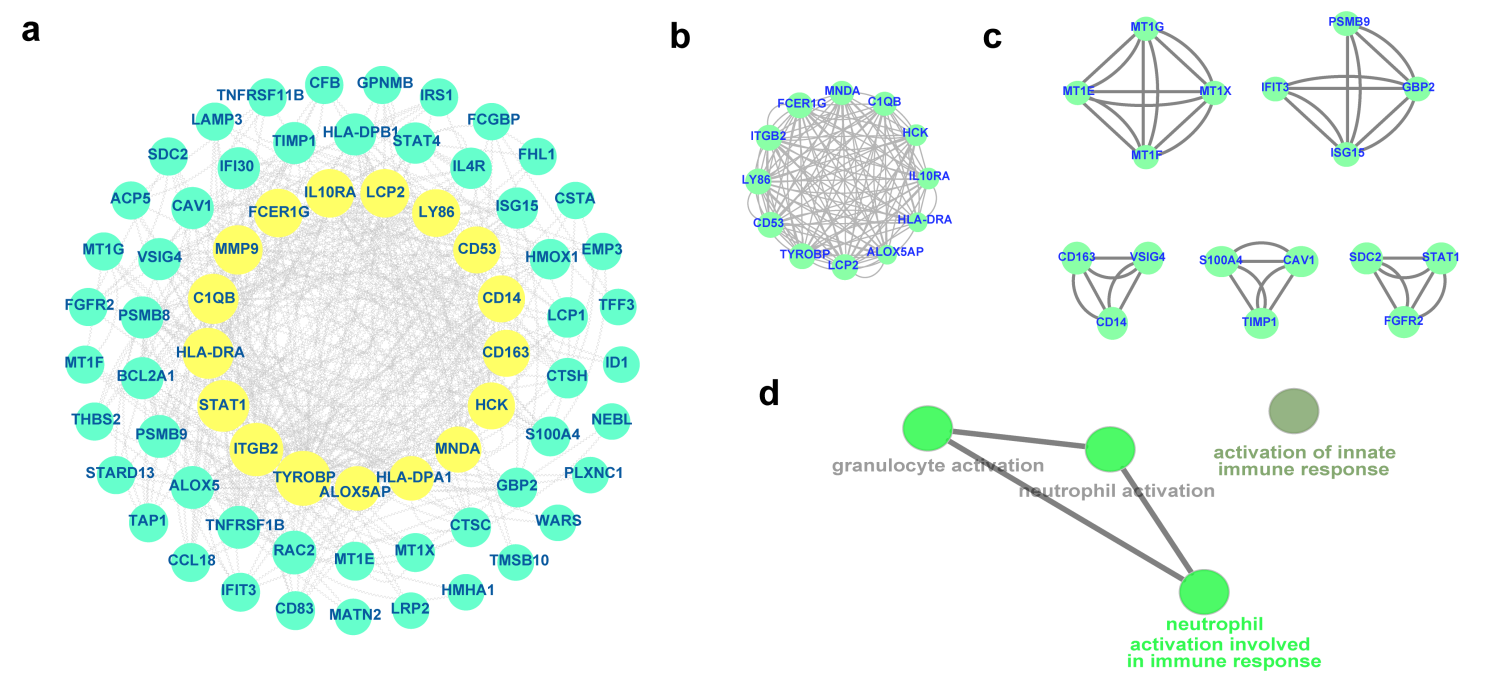
**

**Supplementary Figure 1.** Protein-protein interaction (PPI) network construction and module analysis. **(a)** The PPI network among overlapping DEGs. Node size: the value of DC. **(b)** The most important module of PPI network. **(b,c)** Six cluster modules extracted by MCODE. **(d)** GO enrichment terms of the 12 DEGs in the most important module visualized using the ClueGO plugin.


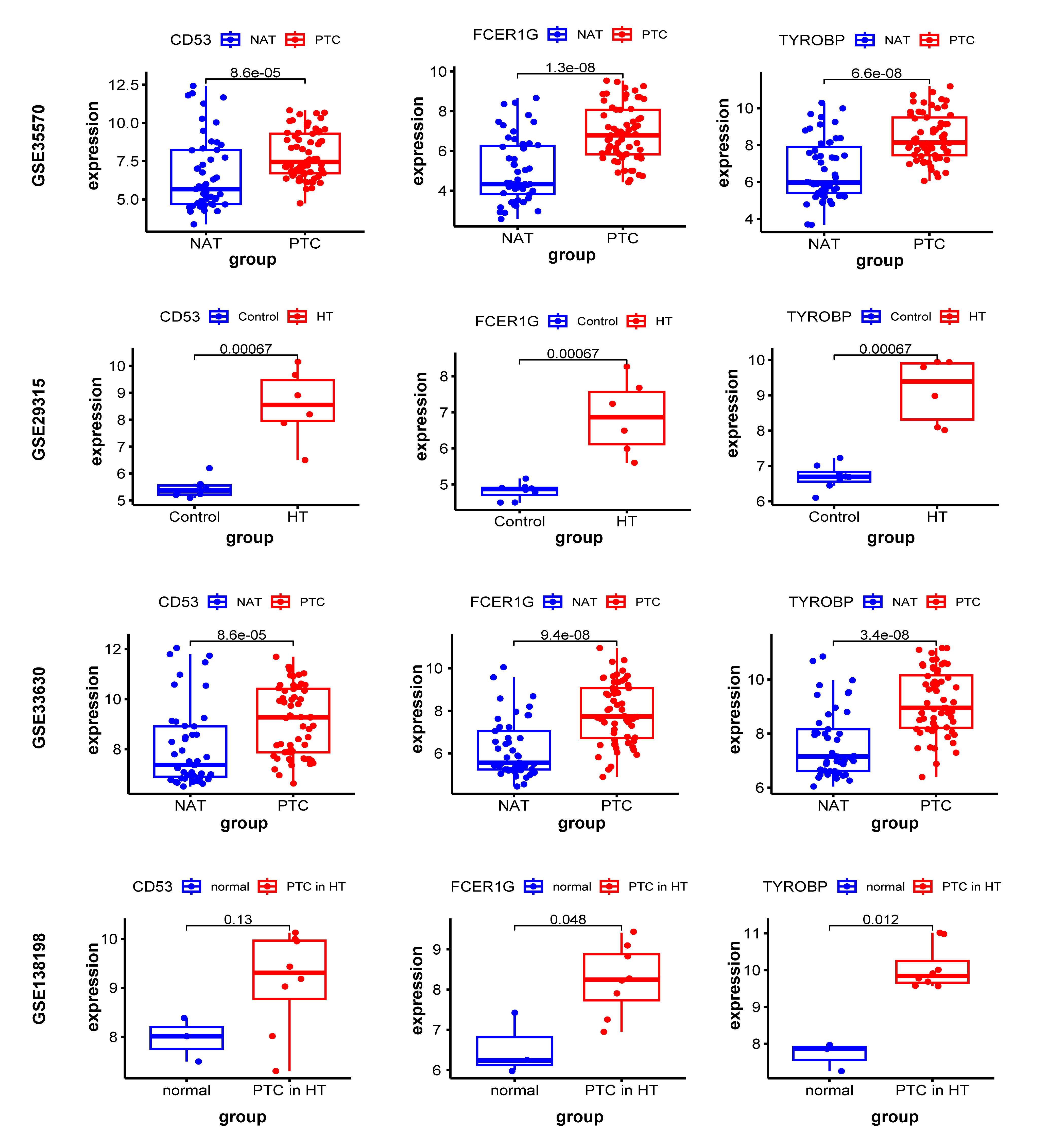


**Supplementary Figure 2.** The expression level of hub genes in GSE35570, GSE29315, GSE33630 and GSE138198.


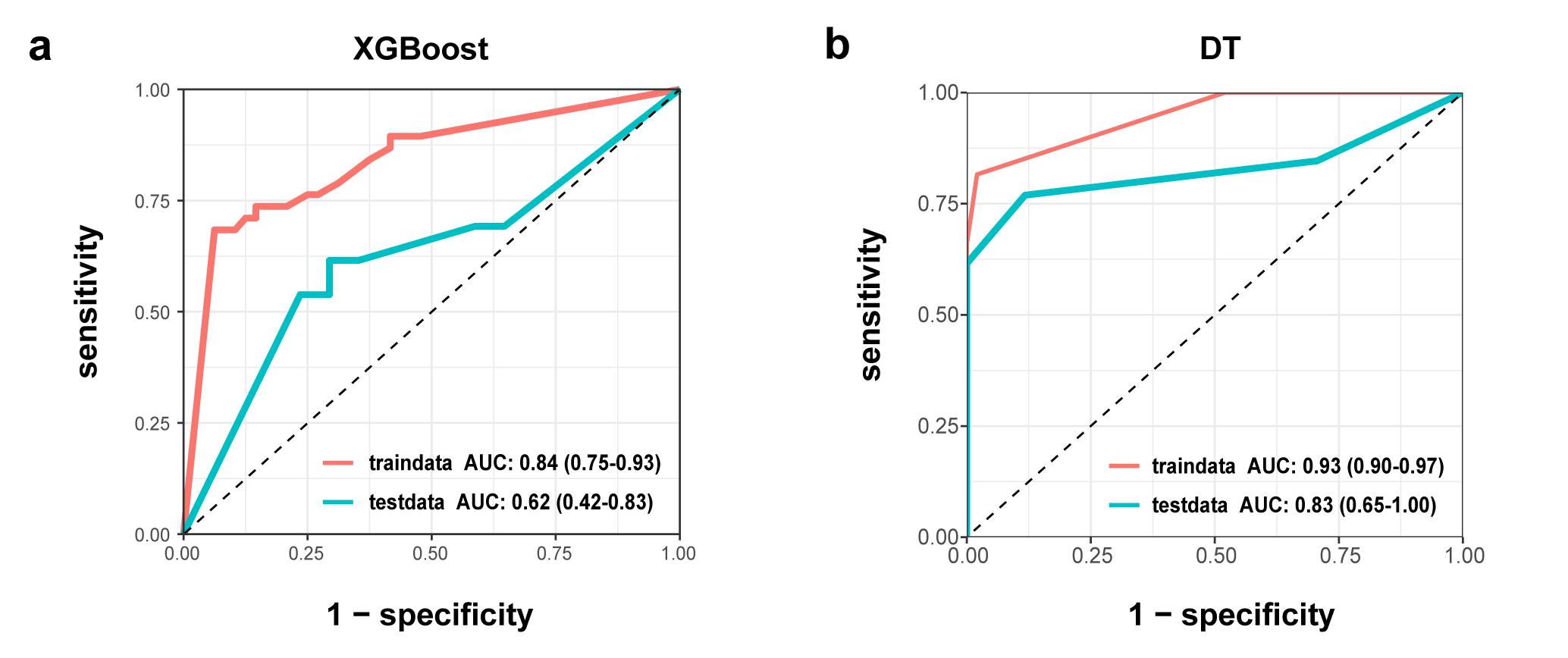


**Supplementary Figure 3.** Model performance evaluation. **(a)** Diagnostic value of the EXtreme Gradient Boosting (XGBoost) model in the GSE35570. **(b)** Diagnostic value of the Decision Tree (DT) model in the GSE35570.


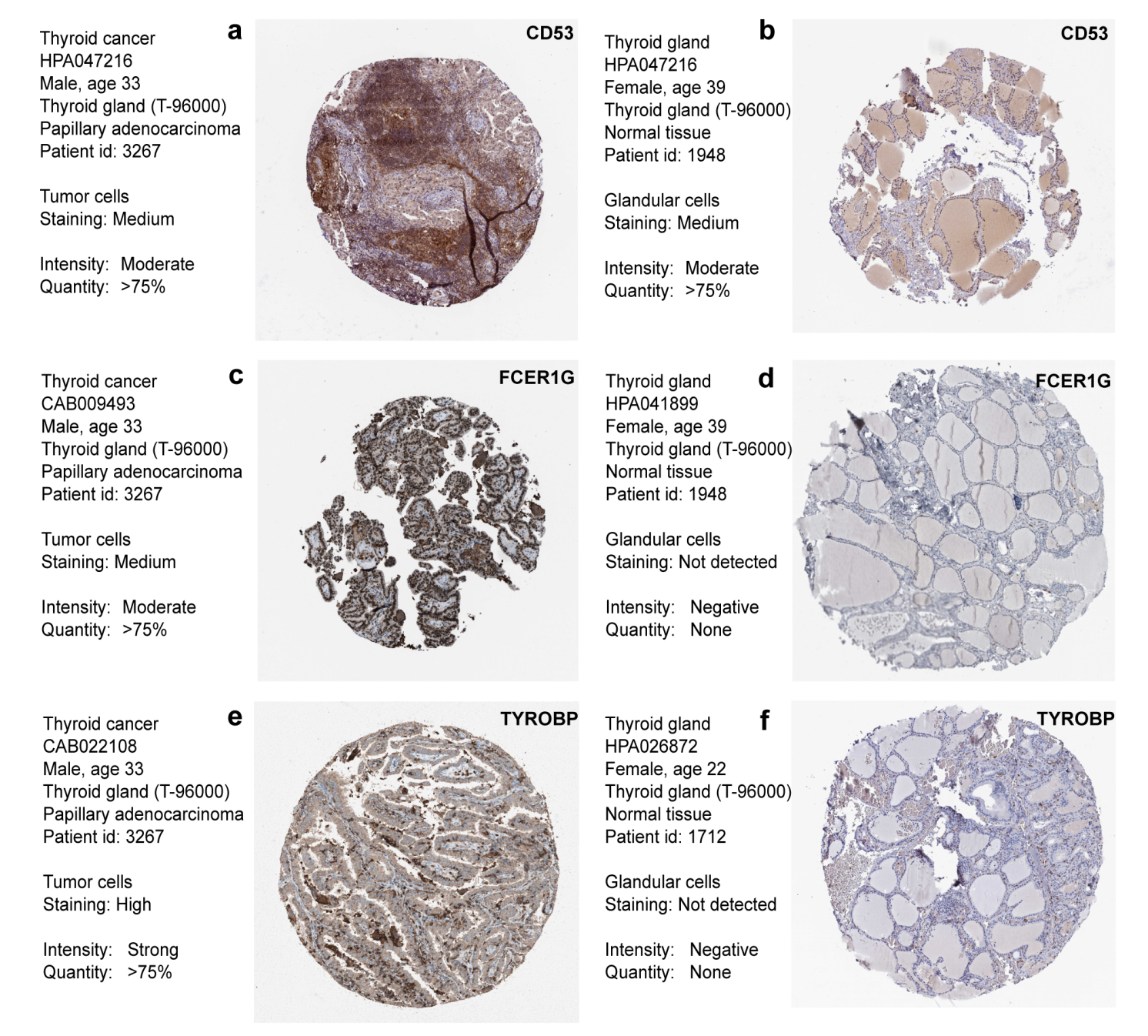


**Supplementary Figure 4.** Gene expression in the HPA database.

**
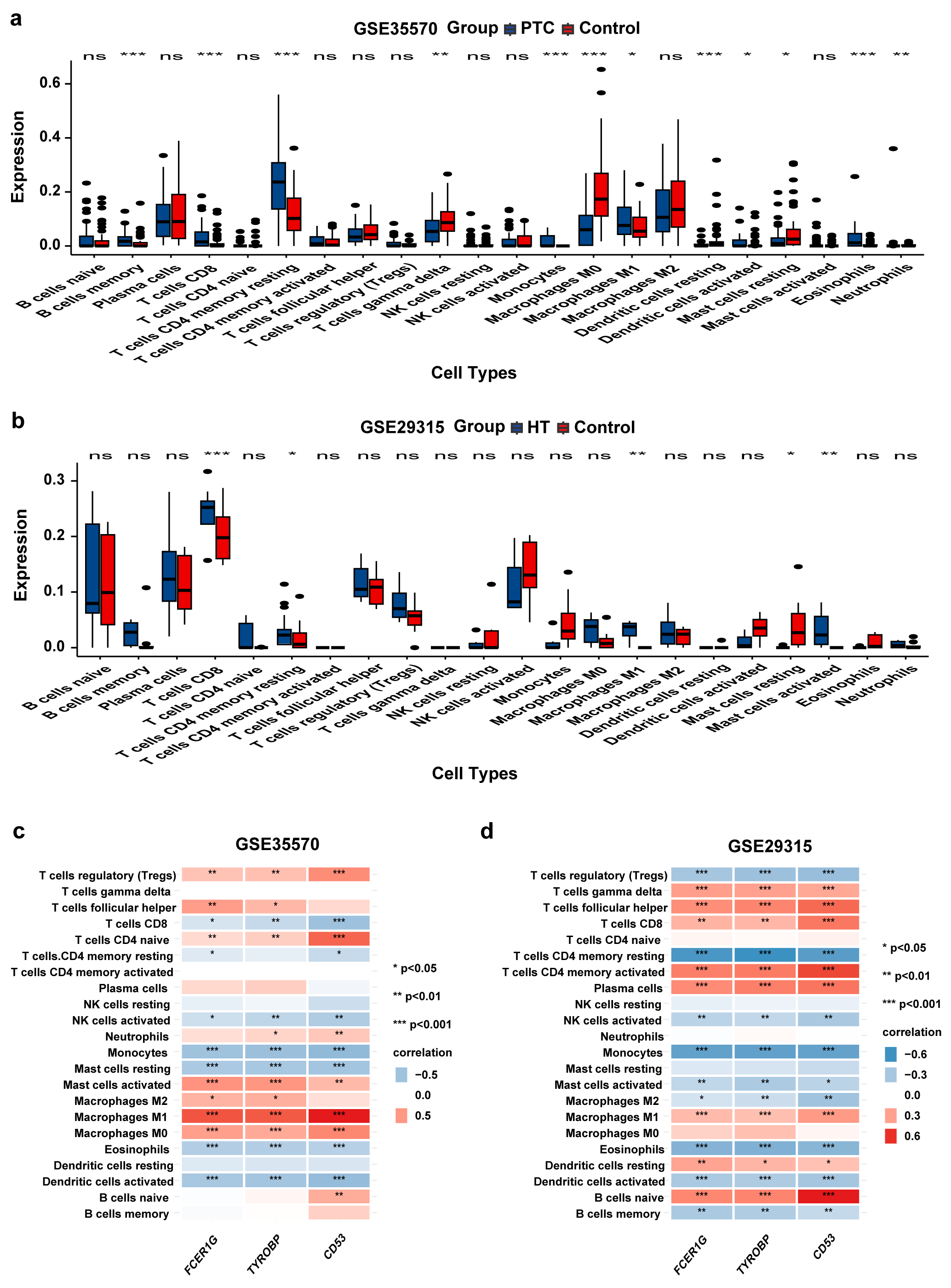
**

**Supplementary Figure 5.** Immune Infiltration analysis. **(a)** Comparison regarding the proportion of 22 kinds of immune cells between PTC and normal groups. **(b)** Comparison regarding the proportion of 22 kinds of immune cells between HT and control groups. **(c)** Correlation between the immune infiltrating cells and hub genes in PTC. **(d)** Correlation between the immune infiltrating cells and hub genes in HT.*P < 0.05; **P < 0.01; ***P < 0.001; NS, not significant.

**Supplementary Table 1.** Diagnostic value of models in the GSE35570.

| **Model** | **Training set** | | **Test set** | |
| --- | --- | --- | --- | --- |
|  | **AUC** | **95%CI** | **AUC** | **95%CI** |
| ANN | 0.94 | 0.91-0.98 | 0.94 | 0.83-1.00 |
| DT | 0.93 | 0.90-0.97 | 0.83 | 0.65-1.00 |
| XGBoost | 0.84 | 0.75-0.93 | 0.62 | 0.42-0.83 |

ANN: Artificial Neural Network; DT: Decision Tree; XGBoost: EXtreme Gradient Boosting.
